# The secretion of cytokines by peripheral blood mononuclear cells of patients with periodontitis and healthy controls when exposed to H_2_S

**DOI:** 10.1080/20002297.2021.1957368

**Published:** 2021-08-10

**Authors:** Amina Basic, Giovanni Serino, Åsa Leonhardt, Gunnar Dahlén, Johan Bylund

**Affiliations:** a Oral Microbiology and Immunology, the Sahlgrenska Academy, University of Gothenburg, Gothenburg, Sweden; b Department of Periodontology, Södra Älvsborgs Hospital, Borås, Sweden

**Keywords:** H_2_S, periodontitis, PBMCs, TNF-α, IFN-γ, IL-6, IL-8, IL-12p40, IL-12p70, IL-17, MCP-1, IL-1Ra

## Abstract

**Background:** Hydrogen sulfide(H_2_S) is a bacterial metabolite produced as a result of bacterial growth in subgingival pockets, suggested to partake in the pathogenesis of periodontitis. H_2_S has previously been shown to induce the secretion of the pro-inflammatory cytokines IL-1β and IL-18 via the NLRP3 inflammasome in monocytes.

**Objective:** To investigate the non-NLRP3 inflammasome-dependent immunological response of human peripheral blood mononuclear cells (PBMCs) of periodontitis patients and healthy controls exposed to H_2_S *in vitro*.

**Methods:** PBMCs of periodontitis patients(N = 31) and healthy controls(N = 32) were exposed to 1 mM sodium hydrosulfide (NaHS) at 37°C for 24 h and the secretion of cytokines was compared to resting cells. TNF-α, IFN-γ, IL-6, IL-8, IL-12p40, IL-12p70, IL-17, MCP-1, and IL-1Ra secretions were measured with Bio-Plex Pro™ Human Cytokine Assay.

**Results:** H_2_S triggered the secretion of the pro-inflammatory IFN-γ, IL-6, IL-17, TNF-α, IL-12p40, and IL-12p70, while the reverse was seen for IL-1Ra. In addition, a higher basal secretion of IFN-γ, IL-6, IL-12p70, IL-17 and MCP-1 was seen from PBMCs of periodontitis patients compared to healthy controls.

**Conclusion:** The bacterial metabolite H_2_S triggers the secretion of pro-inflammatory cytokines from PBMCs and may thus have a prominent role in the host-bacteria interplay in periodontitis.

## Introduction

Periodontitis is an inflammatory disease affecting the supporting tissues of teeth. Bacteria are essential but not sufficient components in the disease etiology. The way the tissue responds to the bacterial load (host susceptibility) is comparably important for the disease development [1]. According to the ecological plaque hypothesis, the disease develops due to an imbalance (dysbiosis) between the bacteria and the host, resulting in a pathological tissue response with loss of periodontal attachment, alveolar bone, and subsequently loss of teeth [2]. Certain anaerobic, proteolytic, and Gram-negative bacterial species have been attributed an important role in disease development [3]. These bacteria are ordinary commensals that adapt and grow in the subgingival environment, favored by the elevated access to proteins, peptides, and amino acids from the gingival crevicular fluid, and the deepening of the periodontal pocket for retention and reduction of the redox-potential. Thus, these microorganisms have, according to the current paradigm, adapted to this ecological niche created due to certain environmental changes, involving anaerobiosis and proteolytic activity, that occur in the periodontal pocket during inflammation [4]. Yet, the same pathogens may also be present in healthy subjects in small numbers and the mechanisms involved in the host-bacteria interaction and pathogenesis of periodontal disease is still not fully determined [1].

It has previously been claimed that it is the activity of the bacteria that is of importance for initiation and progression of periodontitis and other oral diseases, and not necessarily the biofilm composition per se [5,6]. Thus, it is the net effect of all bacteria collectively, usually in a biofilm, that is of significance in the host-bacteria interplay, which determines the well-being of the periodontium. The bacterial metabolites are the end products of bacterial growth and hydrogen sulfide (H_2_S) is such a metabolite suggested to partake in the pathogenesis of periodontitis [7]. During bacterial growth in the periodontal pocket the amino acid cysteine is degraded by bacterial enzymes and H_2_S is, among other substances, formed [8–10]. H_2_S is a small volatile sulfur compound that can brake disulfide bonds, and pass through cell membranes [11]. It was previously known for its environmental toxicity but has in recent years been shown to, mainly depending on the concentration, have several both pro- and anti-inflammatory effects on various host cells [12–14]. The cleavage of disulfide bonds has been suggested to be involved in the inhibition of antibody-mediated immune responses by reduced binding to cell surface antigens [15]. It has also been shown that bacteria are more resistant to leukocyte-mediated killing in the presence of elevated H_2_S levels, but the detailed mechanisms of this protection are not known [16]. Apart from bacterial (exogenous) production, H_2_S is also endogenously produced by the human body and functions as an signaling molecule, both in the brain and at other locations where it can regulate blood pressure, among many other functions [14,17].

We have in a previous *in vitro* study shown that H_2_S can induce the formation of the multiprotein complex NLRP3 inflammasome and the following secretion of IL-1β and IL-18 in human peripheral blood mononuclear cells (PBMCs) and in the human monocyte cell line THP1 [7]. Furthermore, PBMCs of patients with periodontitis have been shown to secrete higher levels of IL-1β and IL-18 compared to healthy controls, both resting cells and cells exposed to H_2_S. These results have led us to hypothesize that H_2_S-producing microorganisms in the subgingival pocket trigger an inflammatory host response by the secretion of pro-inflammatory cytokines and that the level of this secretion may be indicative of the susceptibility of the host to periodontitis.

## Aim

The aim of this study was to investigate the non-NLRP3 inflammasome-dependent immunological response to H_2_S by studying PBMCs of periodontitis patients and healthy controls exposed to a H_2_S donor *in vitro*.

## Material and methods

### Collection of blood samples

This study was approved by the Regional Ethical Review Board (Dnr 871–15) in Gothenburg, Sweden. Detailed information and clinical data of the examined subjects have previously been published [18]. Briefly, the patients diagnosed with periodontitis were examined and recruited at a specialist clinic, Department of Periodontology, Södra Älvsborgs Hospital, Borås, Sweden. Of these, 66% were men with a mean age of 54 years. They had to have at least 14 teeth remaining to be included but presented a mean of 25 teeth remaining, with a mean BoP of 49%. Of the 32 subjects examined, 91% had at least one pocket with a PPD of 7 mm or more. The subjects in the healthy control group were volunteers, staff members at the dental clinics or research personnel, examined and recruited at the Institute of Odontology, University of Gothenburg, Gothenburg, Sweden. In this group there was an equal distribution regarding the gender. These subjects had almost all their teeth remaining, and they had in general a healthy gingiva and no clinical signs of attachment loss due to periodontitis with a mean BoP of 19%. In total 64 subjects were examined and 63 agreed to have peripheral blood samples taken, that were harvested at the examination visit. In the periodontitis group 28% were smokers while none of the subjects in the healthy group smoked. Snuff users were equally represented in both groups. Medical diseases such as high blood pressure and diabetes were reported in both groups but were more common in the periodontitis group [18].

### Isolation of cells from peripheral blood

Peripheral blood mononuclear cells (PBMCs) were isolated by centrifugation over Ficoll-Paque™ Plus density gradients (GE Healthcare Bio-Sciences AB, Uppsala, Sweden) and exposed to a hydrogen sulfide generator as previously described [7,18]. In brief, after a washing step in PBS, the cells were resuspended in Dulbecco’s Modified Eagle Medium + GlutaMAXTM (Gibco, Life Technologies, Paisley, UK) supplemented with 5% human serum (Sigma–Aldrich Sweden AB, Stockholm, Sweden) and penicillin–streptomycin (Invitrogen, Lidingö, Sweden). The cells were seeded, 2 × 10^6^ cells/ml, and cultured in 96-well microtiter plates at 37°C (humidified atmosphere, 5% CO_2_), in the presence or absence of 1 mM sodium hydrosulfide (NaHS; Fisher Scientific). This relatively high concentration was chosen since a concentration of up to 1.9 mM H_2_S previously has been measured from gingival crevicular fluid of deep periodontal pockets [8]. The culture supernatants were collected after 24 h and stored at −80°C until further use.

### Detection of cytokines

The cytokine expressions were measured using a combination of a custom-made premixed x-Plex assay for detection of TNF-α, IFN- γ, IL-6, IL-8, IL-12p70, IL-17, MCP-1, and IL-1R, and a manually added single plex set for detection of IL-12p40 (Bio-Plex Express Assay, Bio-Rad Laboratories, Hemel Hempstead, UK), using Luminex xMAP technology according to the manufacturer’s instructions.

Briefly, all samples were incubated with sets of distinctly color-coded beads conjugated with capture antibodies directed against a specific analyte. After washing, a biotinylated detection antibody was added and allowed to react with the bound proteins. After another washing step, streptavidin conjugated to the fluorescent indicator phycoerythrin was added. Then, the final washing step was followed by acquisition of data using a BioPlex 200 instrument equipped with BioManager analysis software (BioRad). The absolute concentrations of the cytokines were determined by comparing the bead color and mean fluorescence intensity from each set of beads against an automatically optimized and manually verified standard curve. The cytokine concentration was presented as pg/mL.

### Statistical analyses

Statistics were performed using the GraphPad Prism version 7.0 software (GraphPad Inc., La Jolla, CA, USA). The non-parametric Mann–Whitney U-test and Wilcoxon matched-pairs signed-rank test were used for unpaired and paired samples, respectively. A *p*-value <0.05 was considered statistically significant; not significant (ns), *p*< 0.05*, *p*< 0.01**, *p*< 0.001***, *p*< 0.0001****.

## Results

### Cytokine secretion by resting PBMCs from periodontitis patients and healthy controls

**Figure 1.**
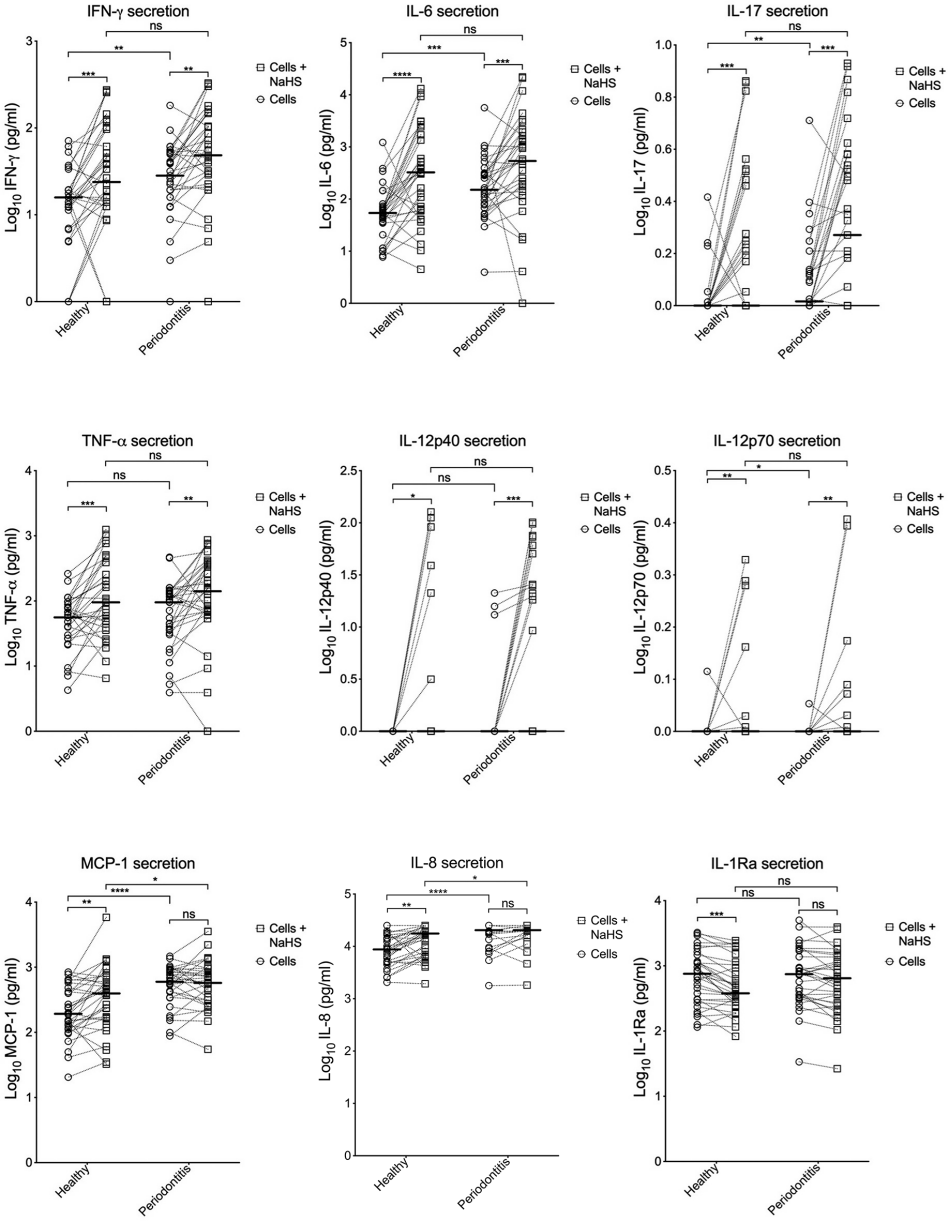
Secretion of cytokines by PBMCs. The secretions of IFN- γ, IL-6, IL-17, TNF-α, IL-12p40, IL-12p70, MCP-1, IL-8, and IL-1Ra of resting PBMCs and PBMCs exposed to NaHS from patients with periodontitis and healthy controls. The median is shown as a vertical line (IFN- γ: 1.20 and 1.39 for healthy, 1.45 and 1.69 for periodontitis. IL-6: 1.73 and 2.51 for healthy, 2.18 and 2.73 for periodontitis. IL-17: 0 and 0 for healthy, 0.02 and 0.27 for periodontitis TNF-α: 1.75 and 1.98 for healthy, 1.98 and 2.15 for periodontitis. IL-12p40: 0 and 0 for healthy, 0 and 0 for periodontitis. IL-12p70: 0 and 0 for healthy, 0 and 0 for periodontitis. MCP-1: 2.28 and 2.60 for healthy, 2.78 and 2.76 for periodontitis. IL-8: 3.83 and 3.78 for healthy, 0 and 0 for periodontitis. IL-1Ra: 2.88 and 2.59 for healthy, 2.87 and 2.81 for periodontitis)

The basal secretion of all cytokines tested is seen in Table 1 and Figure 1. Resting PBMCs from patients with periodontitis secreted more IFN- γ, IL-6, IL-12p70, IL-17 and MCP-1 compared to healthy subjects. This difference was statistically significant. Regarding IL-12p70, resting PBMCs from many of the subjects in both groups secreted undetectable levels. The secretion of IL-8 was statistically significantly higher from PBMCs of periodontitis patients compared to cells from healthy controls but many samples from both groups were above detection limit. There was no difference in secretion of IL-12p40, TNF-α, and IL-1Ra between the two groups. IL-12p40 was, however, not detected in any of samples from healthy subjects and in only a few samples from subjects with periodontitis.

**Table 1.** The median secretion (pg/mL) of the examined markers

Marker	Healthy		*p*-value	Periodontitis		*p*-value
	Cells	Cells±NaHS		Cells	Cells±NaHS	
TNF-α	55.9 (59.1)	94.4 (317)	0.0004	95.3 (101)	141 (298)	0.001
IFN- γ	15.8 (11.2)	24.7 (75.5)	0.0007	28.23 (36.9)	48.5 (82.2)	0.003
IL-6	53.6 (37.2)	320 (1560)	<0.0001	150 (248)	536 (1790)	0.0009
IL-8	8,950 (12,300)	16,500 (18,200)	0.0006	19,700 (8,000)	19,700 (5,400)	0.1040
IL-12p40	0 (0)	0 (0)	0.030	0 (0)	0 (25.3)	0.0005
IL-12p70	0 (0)	0 (0.65)	0.008	0 (0.48)	0.41 (0.91)	0.006
IL-17	0.66 (0.92)	0.91 (2.63)	0.0005	1.03 (0.89)	1.87 (3.11)	0.0001
MCP-1	190 (355)	402 (511)	0.006	603 (547)	573 (640)	0.885
IL-1Ra	753 (1090)	390 (757)	0.0001	746 (1370)	644 (1110)	0.094

The data are presented as medians (interquartile range). Wilcoxon matched-pairs signed-rank test was used to compare the two groups (cells *vs* cells+NaHS).

### Differences in cytokine secretion by resting PBMCs and PBMCs exposed to H_2_S

Exposure of PBMCs to H_2_S triggered statistically significant higher levels of IFN-γ, IL-6, IL-17, and TNF-α secretion, both in the healthy group and in the periodontitis group. A higher secretion by PBMCs exposed to H_2_S was also seen for IL-12p40 and IL-12p70, but many of the subjects showed no detectable secretion, especially by resting cells. The secretion of MCP-1 and IL-8 was higher when the PBMCs were exposed to H_2_S in the healthy group while no statistical difference was seen for cells from patients with periodontitis. IL-1Ra showed a lower secretion by exposed PBMCs compared to resting cells in both groups, but the difference was statistically significant only for the healthy group.

### Cytokine secretion by PBMCs from periodontitis patients and healthy controls exposed to H_2_S

PBMCs from patients with periodontitis exposed to H_2_S secreted statistically significant more MCP-1 compared to healthy controls. No differences were seen for IL-8, since many of the subjects in the periodontitis group had levels above detection. As with resting cells, there was no difference in secretion of IL-12p40, TNF-α, and IL-1Ra between the two groups. Additionally, no statistically significant differences were detected for IFN-γ, IL-6, IL-12p70, and IL-17 for PBMCs exposed to H_2_S.

## Discussion

The main finding of the present study was that H_2_S stimulated the secretion of IFN-γ, IL-6, IL-17, TNF-α, IL-12p40, and IL-12p70, from PBMCs of both periodontitis patients and healthy controls, while the reverse was seen for IL-1Ra, that is, H_2_S decreased the secretion. In addition, a difference in secretion of the majority of the examined cytokines by resting (in the absence of H_2_S) PBMCs of periodontitis patients compared to healthy controls was seen, where higher levels were detected from cells from patients with periodontitis. These results are in line with the results of IL-1β and IL-18 secretion previously investigated in the same cohort [18]. In that study, the capacity of the subgingival microbiota to produce H_2_S was also examined and was shown to be positively correlated to bleeding on probing scores of the patients.

Of the nine proteins measured, the ones triggered by H_2_S, that is, IFN- γ, IL-6, IL-17, TNF-α, IL-12p40, and IL-12p70, are all considered to be part of a pro-inflammatory panel of inflammatory mediators [19]. Together with IL-1β, TNF-α has been extensively studied in relation to periodontitis as a pro-inflammatory and immunomodulatory cytokine [19–21]. IL-12 is generally known for its participation in the differentiation of naïve T-cells to Th1 cells while it has, in relation to periodontitis, been reported to have both pro- and anti-inflammatory properties [22]. Similarly, also IL-6 has been shown to have both pro- and anti-inflammatory properties [23]. IL-6 increases the production of acute-phase proteins in the liver, and has also been linked to bone resorption in periodontitis [20]. In a systematic review and meta-analysis, it was reported that the levels of IL-1β, IFN- γ, MCP-1, and IL-6 in gingival crevicular fluid of periodontitis patients were higher than in healthy individuals, while no differences were observed for IL-12 and IL-17 [21]. Further, it was reported that the levels of IL-1β and IL-17 significantly decreased after periodontal treatment, while another systemic review reported reduced levels of, among others, IL-1β, IL-6, TNF-α, IFN- γ, and MCP-1, six to eight weeks after periodontal treatment [24]. Thus, the inflammatory mediators measured in our study have previously been linked to periodontitis. The potential role of the bacterial metabolite H_2_S as a critical initiator and maintainer of this host response is, however, a novelty. Collectively, the measurement of several cytokines conducted in the present study shows a distinct pro-inflammatory pattern from PBMCs exposed to H_2_S, while the anti-inflammatory IL-1Ra (IL-1 receptor antagonist) which binds to the same receptor as IL-1β, is decreased [25].

A higher basal secretion from PBMCs of patients with periodontitis compared to healthy controls was in the present study seen for IFN- γ, IL-6, IL-12p70, IL-17, and MCP-1, showing some similarities with the results reported from measurements in gingival crevicular fluid in the aforementioned systematic review and meta-analysis that compared periodontitis patients and healthy controls [21]. Also, a statistically significantly higher secretion by resting PBMCs of the present cohort was seen for IL-1β in patients with periodontitis while there was no difference between periodontitis patients and healthy controls regarding the acute-phase protein CRP, as previously reported [18]. A lack of difference regarding the CRP levels has also been reported by other studies, while the more sensitive serum amyloid A has been shown to be detected in higher levels in periodontitis patients compared to healthy controls [26,27]. Taken together, the data suggests that a higher basal secretion of pro-inflammatory mediators in periodontitis-susceptible individuals may be the result of a systemic inflammatory process.

In periodontitis, the host response to the colonizing bacteria is of pivotal importance for disease progression, especially considering the fact that the various bacterial species harboring the subgingival pocket are commensals [1]. Thus, all individuals are colonized with various oral bacterial species that can adapt to a subgingival environment and that have the capacity to produce H_2_S [9,10,28]. Nevertheless, only a fraction of approximately 10% develops severe periodontitis, while other individuals never proceed from gingivitis [29]. Although, H_2_S can trigger the secretion of pro-inflammatory cytokines from both PBMCs of periodontitis patients and healthy controls, the differences between these two groups of patients, that is, the secretion of MCP-1 and IL-8, may be indicative of differences in host response between the periodontitis susceptible and periodontitis unaffected subjects. MCP-1 and IL-8 were secreted in higher levels from PBMCs of periodontitis patients. Both proteins are chemokines, MCP-1 is recruiting monocytes and T lymphocytes, while IL-8 activates neutrophils [19,30]. It should, however, be noted that the secreted IL-8 levels detected in our study were either generally high both from resting and exposed PBMCs in both groups or above the level of detection. The potential meaning of these observations needs to be addressed in future studies.

H_2_S produced by bacterial growth may not be the only source to its presence in the periodontal pocket. H_2_S is also endogenously produced and also macrophages possess the enzymes for H_2_S production [17]. Thus, on one side the presence of H_2_S in the periodontal pocket may be interpreted as positive for the bacteria in the bacteria-host interplay since H_2_S can induce the secretion of pro-inflammatory cytokines of the host, dampen leukocyte-mediated killing of bacteria, and promote bacterial resistance to antibiotics [6,16,31]. Consequently, the subgingival bacteria are favored by the presence of H_2_S. On the other hand, however, are the reports of endogenously produced H_2_S that in low concentrations is initiating secretion of anti-inflammatory cytokines, and is thus beneficial for the host [17]. It is inarguable this fact, that H_2_S is recognized as an endogenous substance, that potentially can confuse the host inflammatory response. Accordingly, the *in vivo* situation in the periodontal pocket is complex and the role of H_2_S is difficult to interpret. The biphasic properties of H_2_S trigger the inflammatory response of the host and may simultaneously suppress the clearance of the infection, thus contributing to the never-ending bacteria-host interplay that is characteristic for periodontitis.

## Conclusion

The inflammatory host response to bacteria is essential in the pathogenesis of periodontitis. In this study, we report on the effect of the bacterial metabolite H_2_S on the secretion of inflammatory mediators from mononuclear leukocytes *in vitro*. Exposure to H_2_S triggers the secretion of pro-inflammatory cytokines. In addition, some differences in the secretory response of cells from periodontitis patients and healthy controls are reported. Further studies are needed to assess H_2_S as a potential virulence factor in the pathogenesis of periodontitis.
