# Correction

**DOI:** 10.1080/20002297.2024.2393043

**Published:** 2024-08-20

**Article title**: The secretion of cytokines by peripheral blood mononuclear cells of patients with periodontitis and healthy controls when exposed to H_2_S

**Authors**: Amina Basic, Giovanni Serino, Åsa Leonhardt, Gunnar Dahlén and Johan Bylund

**Journal**: *Journal of Oral Microbiology*

**Bibliometrics**: Volume 11, Number 01, pages 1-7

DOI: https://doi.org/10.1080/20002297.2021.1957368

It has been noted by the authors that the image of **IL-8** in Figure 1 and Table 1 were incorrect in the published article. The corrected image of **IL-8** in Figure 1 and Table 1 have been placed below. This correction has not changed the description, interpretation, or the original conclusions of the article. The authors apologize for any inconvenience caused.

**Figure 1.**
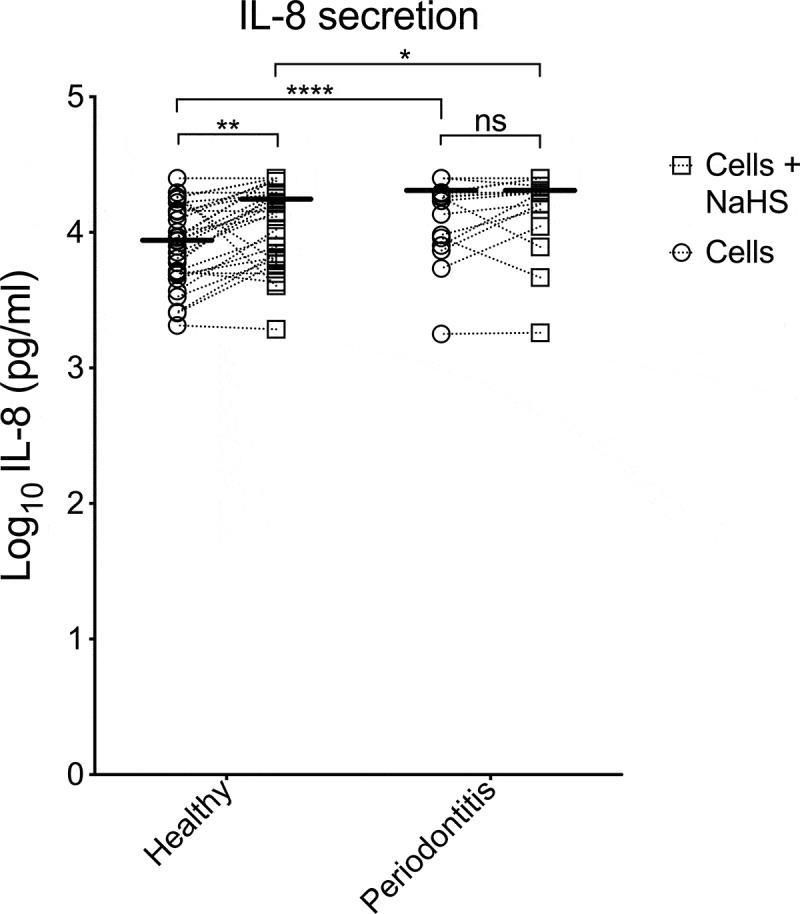

**Table 1.** The median secretion (pg/mL) of the examined markers.

Marker	Healthy		p-value	Periodontitis		p-value
	Cells	Cells+NaHS		Cells	Cells+NaHS	
TNF-α	55.9 (59.1)	94.4 (317)	0.0004	95.3 (101)	141 (298)	0.001
IFN-γ	15.8 (11.2)	24.7 (75.5)	0.0007	28.23 (36.9)	48.5 (82.2)	0.003
IL-6	53.6 (37.2)	320 (1560)	<0.0001	150 (248)	536 (1790)	0.0009
IL-8	8 950 (12 300)	16 500 (18 200)	0.0006	19 700 (8 000)	19 700 (5 400)	0.1040
IL-12p40	0 (0)	0 (0)	0.030	0 (0)	0 (25.3)	0.0005
IL-12p70	0 (0)	0 (0.65)	0.008	0 (0.48)	0.41 (0.91)	0.006
IL-17	0.66 (0.92)	0.91 (2.63)	0.0005	1.03 (0.89)	1.87 (3.11)	0.0001
MCP-1	190 (355)	402 (511)	0.006	603 (547)	573 (640)	0.885
IL-1Ra	753 (1090)	390 (757)	0.0001	746 (1370)	644 (1110)	0.094

The data are presented as medians (interquartile range). Wilcoxon matched-pairs signed-rank test was used to compare the two groups (cells *vs* cells+NaHS).

